# Intraoperative assessment and reporting of radical prostatectomy specimens to guide nerve‐sparing surgery in prostate cancer patients (NeuroSAFE)

**DOI:** 10.1111/his.14184

**Published:** 2020-09-03

**Authors:** Margaretha A van der Slot, Michael A den Bakker, Sjoerd Klaver, Mike Kliffen, Martijn B Busstra, John B W Rietbergen, Melanie Gan, Karen E Hamoen, Leo M Budel, Natascha N T Goemaere, Chris H Bangma, Jozien Helleman, Monique J Roobol, Geert J L H van Leenders

**Affiliations:** ^1^ Anser Prostate Clinic Maasstad Hospital Rotterdam The Netherlands; ^2^ Department of Pathology Maasstad Hospital Rotterdam The Netherlands; ^3^ Department of Urology Maasstad Hospital Rotterdam The Netherlands; ^4^ Department of Urology Erasmus MC University Medical Centre Rotterdam The Netherlands; ^5^ Department of Urology Franciscus Gasthuis & Vlietland Rotterdam The Netherlands; ^6^ Department of Pathology Erasmus MC University Medical Centre Rotterdam The Netherlands

**Keywords:** frozen section, NeuroSAFE, prostate cancer, prostatectomy, surgical margin

## Abstract

**Aims:**

Radical prostatectomy for prostate cancer is frequently complicated by urinary incontinence and erectile dysfunction. Nerve‐sparing surgery reduces the risk of postoperative complications and can be optimised by the use of intraoperative frozen sections of the adjacent neurovascular structure (NeuroSAFE). The aims of this study were to evaluate the pathological outcomes of the NeuroSAFE technique and to develop a comprehensive algorithm for intraoperative clinical decision‐making.

**Methods and results:**

Between September 2018 and May 2019, 491 NeuroSAFE procedures were performed in 258 patients undergoing radical prostatectomy; 74 of 491 (15.1%) NeuroSAFE specimens had positive surgical margins. As compared with the corresponding paraffin sections, NeuroSAFE had a positive predictive value and negative predictive value of 85.1% and 95.4%, respectively. In 72.2% of secondary neurovascular bundle resections prompted by a NeuroSAFE positive surgical margin, no tumour was present. These cases more often had a positive surgical margin of ≤1 mm (48.7% versus 20.0%; *P* = 0.001) and only one positive slide (69.2% versus 33.3%; *P* = 0.008). None of the nine patients with Gleason pattern 3 at the surgical margin, a positive surgical margin length of ≤1 mm and one positive slide had tumour in the secondary resection.

**Conclusions:**

This study provides a systematic reporting template for pathological intraoperative NeuroSAFE evaluation, supporting intraoperative clinical decision‐making and comparison between prostate cancer operation centres.

## Introduction

Radical prostatectomy (RP) is one of the main treatment modalities for patients with localised prostate cancer. Although RP was initially mostly performed for low‐risk to intermediate‐risk disease, patients with high‐risk cancer are increasingly being offered RP in Europe and North America.^1^, ^2^ Despite its efficacy in oncological disease control, RP is complicated by urinary incontinence and erectile dysfunction in 3–16% and 20–90% of patients, respectively.^3^, ^4^ Surgical preservation of neurovascular bundles adjacent to the prostate, urologists' experience and centralisation in high‐volume expert centres can all contribute to reducing complication rates.^5^, ^6^, ^7^, ^8^, ^9^ Clinical suspicion of extraprostatic expansion is a relative contraindication for nerve‐sparing surgery, limiting its potential beneficial effects in this high‐risk subgroup of prostate cancer patients.^10^, ^11^, ^12^

Standardised intraoperative frozen section (IFS) assessment of surgical margins during RP according to the NeuroSAFE technique has been shown to significantly increase nerve‐sparing surgery without negatively affecting oncological outcome.^9^, ^13^, ^14^, ^15^ For this purpose, urologists initially perform bilateral nerve‐sparing RP, after which prostate tissue adjacent to the neurovascular bundles, which are still *in situ*, is removed from the specimen and submitted for detailed pathological intraoperative evaluation. If adenocarcinoma does not reach into the surgical margin, the ipsilateral nerve bundle remains intact; in the case of a positive IFS surgical margin, the adjacent neurovascular bundle is then removed. NeuroSAFE is increasingly being offered to prostate cancer patients in Europe.^13^, ^15^, ^16^ Implementation of the NeuroSAFE methodology requires standardisation of pathological evaluation and reporting, and the development of clinical algorithms for subsequent surgical decision‐making.^17^

Since September 2018, seven medical centres in The Netherlands have collaborated within the Anser Prostate Network, in which all RPs are performed with NeuroSAFE in one high‐volume operation clinic. The aims of this study were to report pathological outcomes of the NeuroSAFE technique, and to develop a comprehensive algorithm for pathological reporting and intraoperative clinical decision‐making.

## Materials and methods

### Study Population

Patients undergoing RP for prostate cancer in the Anser Prostate Operation Clinic, Maasstad Hospital, Rotterdam, The Netherlands between September 2018 and May 2019 were included. Intraoperative assessment of surgical margin status according to the NeuroSAFE methodology was offered to the vast majority of patients. NeuroSAFE was not applied unilaterally or bilaterally in cases of clinical T3 disease, fibrotic adhesions, e.g. due to previous operations, or patient anxiety. The study was approved by the local ethics committee (METC‐2019‐0352).

### NeuroSAFE Procedure

NeuroSAFE was performed as described by Schlomm *et al*.^13^ Initially, each patient underwent bilateral nerve‐sparing RP. After removal of the prostate, the urologist cleaved the posterolateral sides adjacent to the neurovascular bundles from apex to base; the neurovascular bundles themselves remained *in situ* during this procedure. The cleaved posterolateral prostate tissues were inked at the apical, outer and inner surfaces for orientation. The right‐inked and left‐inked prostate tissues were submitted for IFS assessment at the pathology department.

If IFS assessment did not reveal tumour in the surgical margin, the operation was finished, leaving the neurovascular bundles intact. If tumour was identified within the IFS surgical margin, partial or total secondary resection of the ipsilateral neurovascular bundle was performed. Partial secondary resection was performed only if the surgeon was able to precisely identify the anatomical area directly adjacent to the positive surgical margin and if the margin was positive in one or, at most two, adjacent slides. The location was determined as the slice number counted from the marked apex.

From September 2018 to February 2019, secondary resection of the neurovascular bundle was performed in all cases with positive IFS surgical margins. After February 2019, secondary resection was performed only if a positive surgical margin was present in more than one slide, had a cumulative length of >1 mm, or contained Gleason pattern 4 or 5 tumour. In secondary bundle resections, the non‐prostate side, i.e. the external surface representing the definitive surgical margin, was inked by the urologist for orientation.

### Frozen Section Analysis

After gross reporting, the inked prostate tissue was transversely cut into 5‐mm sections, resulting in 7–10 slices per side, which were oriented from apex to base. Standard 5‐μm haematoxylin and eosin (H&E)‐stained frozen sections were prepared from the prostate slices. Five pathologists with an interest in genitourinary pathology reported all IFSs. A positive surgical margin was defined as at least one malignant gland abutting the inked margin. In the case of a positive surgical margin, the urologist was informed about the number and location of the positive slides, the cumulative positive surgical margin length, and the Gleason pattern at the margin. After IFS evaluation, the remaining tissue was thawed, formalin‐fixed, and embedded for preparation of standard H&E‐stained slides.

### Pathological Analysis

After formalin fixation, residual RP specimens were transversely sectioned into 4‐mm slices from apex to base, and submitted in their entirety for diagnostic purposes, together with the IFS and formalin‐fixed paraffin‐embedded (FFPE) NeuroSAFE slices. In cases of a secondary neurovascular excision, the tissue was transversely cut into 2‐mm sections. At microscopic evaluation, the following parameters were recorded: Gleason score and Grade Group (GG) according to the World Health Organization 2016 guidelines, pT stage (American Joint Committee on Cancer 8th edition), and surgical margin status. Pathological stage T3a was defined as the presence of prostate cancer cells within or at the level of periprostatic fat tissue. A positive IFS surgical margin without a secondary resection and no extraprostatic extension was defined as pT2. Patients with a positive IFS surgical margin but a negative outer surface margin on secondary resection were considered to have a negative surgical margin in definitive reporting. Patients with a negative IFS surgical margin but a positive surgical margin on the corresponding paraffin section were considered to have a positive surgical margin.

### Statistical Analysis

The median cumulative lengths of positive surgical margins were compared by use of the Mann–Whitney test. Categorical GGs, positive surgical margin length groups and number of positive slides were compared by use of the chi‐square test. The Spearman coefficient was used to determine the association between IFS pathology and operation duration in relation to time. Statistical analyses were performed with IBM spss version 24. A *P*‐value of ≤0.05 was considered to be statistically significant.

## Results

### Patient Characteristics

Between September 2018 and May 2019, 276 men underwent robot‐assisted RP; NeuroSAFE was applied in 258 of these (Table 1). The median age of the 258 patients was 67.0 years [interquartile range (IQR) 63.0–71.0 years], and the median preoperative prostate‐specific antigen level was 9.4 ng/ml (IQR 6.4–12.7 ng/ml). Fifty patients (19.4%) had preoperative biopsy GG1 disease, 101 (39.1%) had GG2 disease, 62 (24.0%) had GG3 disease, 27 (10.5%) had GG4 disease, and 17 (6.6%) had GG5 disease; GG was unknown for one (0.4%). NeuroSAFE was performed bilaterally in 233 (90.3%) patients and unilaterally in 25 (9.7%) patients, resulting in a total of 491 IFS analyses. If NeuroSAFE was performed on one side, the other side was operated on without nerve sparing.

**Table 1:** Preoperative patient characteristics

Parameters	NeuroSAFE patients
Number of patients	258
Age (years), median (IQR)	67.0 (63.0–71.0)
Preoperative PSA (ng/ml), median (IQR)	9.4 (6.4–12.7)
Preoperative Grade Group, *n* (%)	
1	50 (19.4)
2	101 (39.1)
3	62 (24.0)
4	27 (10.5)
5	17 (6.6)
Unknown	1 (0.4)
Total biopsy number, median (IQR)	10.0 (8.0–11.0)
Positive biopsy number, median (IQR)	4.0 (2.0–6.0)
D'Amico risk stratification, *n* (%)	
Low	30 (11.6)
Intermediate	159 (61.6)
High	68 (26.4)
Unknown	1 (0.4)

IQR, Interquartile range; PSA, Prostate‐specific antigen.

### IFSs and Corresponding Paraffin Slides

**Figure 1:**
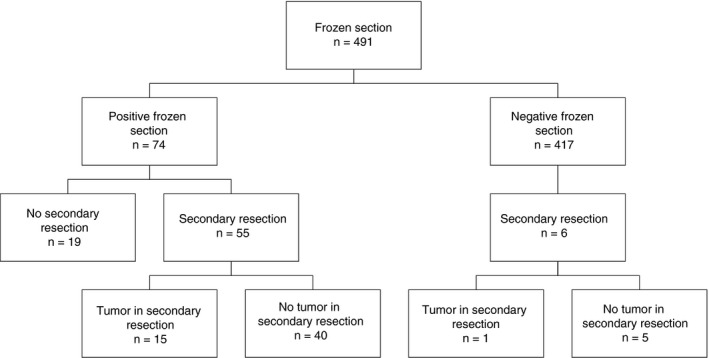
Schematic overview of NeuroSAFE procedures and secondary neurovascular bundle resections.

**Figure 2:**
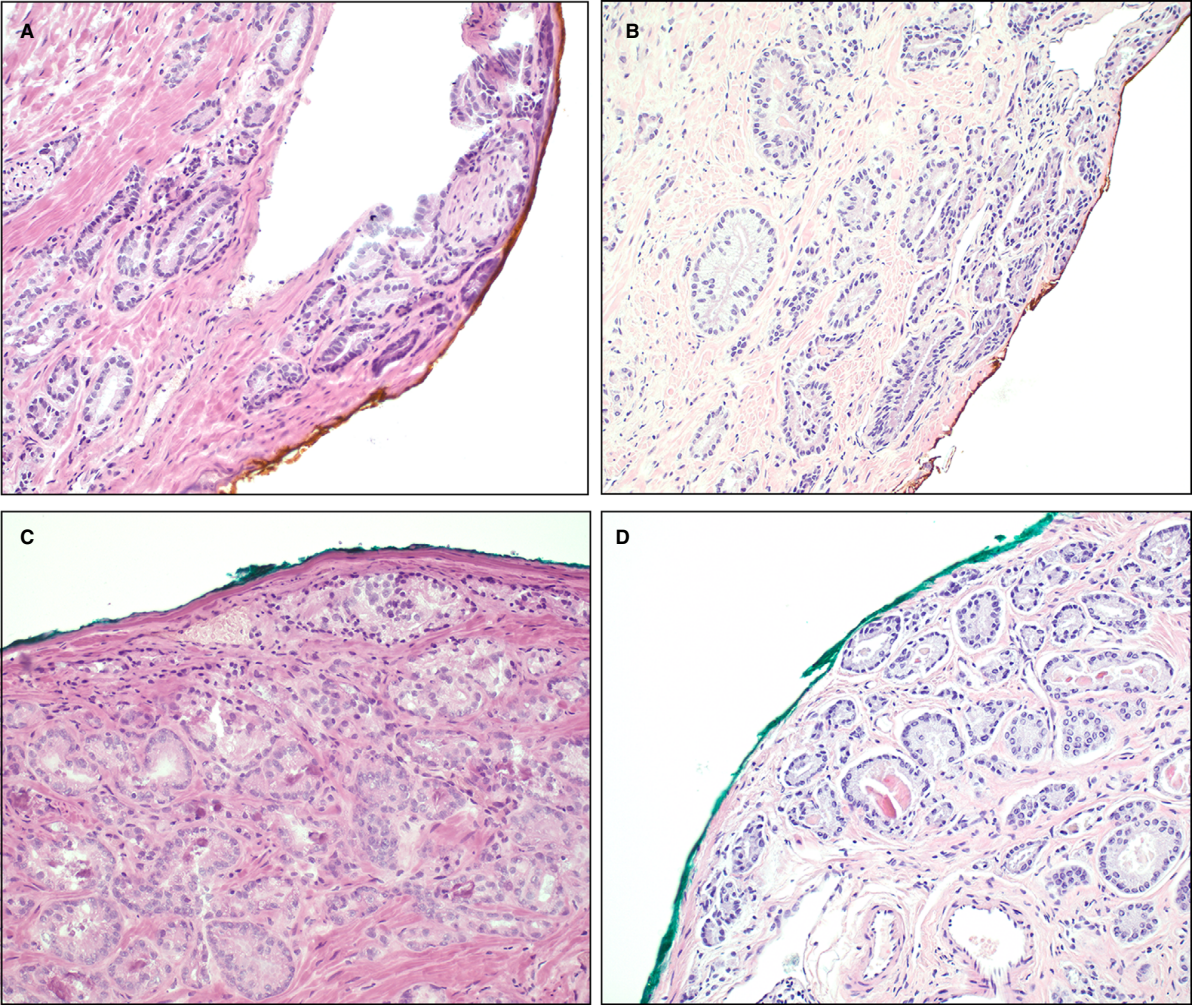
Frozen sections (**A**,**C**) and corresponding paraffin sections (**B**,**D**) of two NeuroSAFE slices. **A**,**B**, Both frozen and corresponding paraffin sections have positive surgical margins with tumour cells into the ink (concordant). **C**,**D**, A frozen section was called surgical margin‐negative, whereas the corresponding paraffin section showed a positive surgical margin (discordant). Haematoxylin and eosin.

Of the 491 NeuroSAFE samples, 417 (84.9%) had negative IFS surgical margins and 74 (15.1%) had positive IFS surgical margins (Figure 1). Corresponding paraffin sections of the IFS slides with negative surgical margins showed a similar surgical margin status in 398 of 417 samples (95.4%; negative predictive value), but were positive in 19 (4.6%) cases (Figure 2). Re‐evaluation of the original IFS slides confirmed negative IFS surgical margins in all 19 cases, indicating that the positive surgical margins in the paraffin sections resulted from deeper cutting of the tissue block. These 19 discrepant cases had a median cumulative positive surgical margin length of 0.2 mm (IQR 0.1–0.4 mm) in the corresponding paraffin sections; 17 of 19 (89.5%) had a positive surgical margin length of ≤1 mm, and two (10.5%) had a positive surgical margin length of between 1 and 3 mm (Table 2).

**Table 2:** Pathological characteristics of intraoperative NeuroSAFE in relation to corresponding paraffin sections and secondary neurovascular bundle resections

Parameter	Frozen and paraffin sections			Secondary resection		
	Positive/positive*	Negative/positive*	*P*‐value	Tumour	No tumour	*P*‐value
NeuroSAFE number	63	19		15	39	
Margin length (mm), continuous^†^	1.1 (0.4–2.4)	0.2 (0.1–0.4)	<0.001	1.6 (1.1–2.2)	1.2 (0.3–3.1)	0.524
Margin length, categorical (mm), *n* (%)						
≤1	30 (47.6)	17 (89.4)	0.012	3 (20.0)	19 (48.7)	0.002
1–2	16 (25.4)	1 (5.3)		8 (53.3)	6 (15.4)	
2–3	6 (9.5)	1 (5.3)		2 (13.3)	4 (10.3)	
>3	11 (17.5)	0		2 (13.3)	10 (25.6)	
Margin Grade Group, *n* (%)						
1	32 (50.8)	11 (57.9)	0.864	10 (66.7)	16 (41.0)	0.025
2	10 (15.9)	2 (10.5)		1 (6.7)	8 (20.5)	
3	7 (11.1)	1 (5.3)		4 (26.7)	2 (5.1)	
4	10 (15.9)	3 (15.8)		0	10 (25.6)	
5	4 (6.3)	2 (10.5)		0	3 (7.7)	
Number of positive slides, *n* (%)						
1	39 (61.9)	18 (94.7)	0.023	5 (33.3)	27 (69.2)	0.004
2	17 (27.0)	1 (5.3)		5 (33.3)	10 (25.6)	
≥3	7 (11.1)	0		5 (33.3)	2 (5.1)	

* Margin of the frozen section/margin of the corresponding paraffin section.

^†^ Median positive surgical margin length (interquartile range).

Corresponding paraffin sections of the 74 samples with positive IFS surgical margins showed concordant positive surgical margins in 63 samples (85.1%; positive predictive value) and were negative in 11 (14.9%) cases (Figure 2). Re‐evaluation of the original IFS slides confirmed positive surgical margins in nine of 11 (81.8%) patients, again indicating that the discrepancy was caused by the inherent 250–300‐µm deeper sectioning of the paraffin block. However, in two samples, re‐evaluation showed that the artificial margin at the prostate slice edge, which was close to the true margin, was erroneously called positive; one of these patients underwent a secondary resection. Therefore, the overall sensitivity and specificity of the IFS as compared with the corresponding paraffin section were 76.8% (63/82) and 97.3% (398/409), respectively.

The median interval from specimen submission to reporting by the pathology department was 43 min (IQR 39–50 min) and decreased over time (Spearman rho −0.26; *P* < 0.001). The median pathology time for the first 100 NeuroSAFE procedures was 46 min (IQR 40–53 min), and that for the last 100 was 41 min (IQR 37–48 min) (*P* = 0.004). For unilateral NeuroSAFE, the median time was 40 min (IQR 31–46 min); for bilateral procedures, it was 44 min (IQR 39–50 min) (*P* = 0.003).

### Secondary Neurovascular Bundle Resections

In 61 of 491 (12.4%) NeuroSAFE procedures, a secondary neurovascular bundle resection was performed. Fifty‐five (90.2%) of these were prompted by a positive IFS surgical margin, and six (9.8%) were performed in spite of a negative IFS surgical margin because of a strong clinical suspicion of extensive disease during operation (Figure 1). Two of these six negative IFS cases had positive margins in the corresponding paraffin slides; in one sample, tumour was present in the secondary resection; in the other five secondary resections, no tumour was found. Fifty‐five of 74 (74.3%) samples with positive IFS surgical margins underwent a secondary resection, but it was omitted in 19 (25.7%) cases, because the margin was only minimally affected by Gleason pattern 3 disease. The two false‐positive IFS cases, one of which had undergone a secondary resection, were excluded from further analysis.

In 15 of 54 (27.8%) secondary resections, adenocarcinoma was present within the neurovascular bundle tissue, whereas, in 39 of 54 (72.2%) cases, no tumour was present. Tumour was present in five of 25 (20.0%) partial secondary resections and in 10 of 29 (34.5%) total secondary resections. Cases without tumour present in the secondary resection more often had a positive IFS surgical margin of ≤1 mm (48.7% versus 20.0%; *P* = 0.001) and only one single surgical margin‐positive IFS slide (69.2% versus 33.3%; *P* = 0.008) than cases with tumour present in the secondary resection (Table 2). In none of the nine NeuroSAFE samples with a positive surgical margin of ≤1 mm and Gleason pattern 3 in one IFS was adenocarcinoma present in the secondary resection. In 51 of 54 (94.4%) NeuroSAFE samples, there was conversion of the definitive neurovascular bundle surgical margin to negative in the secondary resection; two patients had positive surgical margins in secondary resections. Of these, one patient had a unilateral positive surgical margin in a total secondary resection, and one had bilateral positive surgical margins in both partial secondary resections.

### Definitive RP Findings

At final pathological evaluation of the RP specimens, 26 (10.1%) patients had GG1 disease, 115 (44.6%) had GG2 disease, 88 (34.1%) had GG3 disease, 12 (4.7%) had GG4 disease, and 17 (6.6%) had GG5 disease (Table 3). In total, 140 (54.3%) patients had pT2, 79 (30.6%) had pT3a, and 39 (15.1%) had pT3b disease. After analysis of the corresponding NeuroSAFE paraffin sections, RP specimens, and secondary neurovascular bundle resections, 89 of 258 (34.5%) patients had positive surgical margins at final pathology reporting. The margin was positive on the apical, basal or anterolateral non‐NeuroSAFE side in 54 (60.7%) patients, on the posterolateral NeuroSAFE side in 18 (20.2%) patients, and on both the NeuroSAFE side and the non‐NeuroSAFE side in 17 (19.1%) patients. Determination of a final positive margin on a NeuroSAFE side was prompted by: (i) the presence of a positive surgical margin in the corresponding paraffin section of a negative IFS (*n* = 15); (ii) a minutely positive IFS without a secondary resection (*n* = 16); (iii) a positive paraffin margin in a negative IFS on one side and omission of a secondary resection on the contralateral side (*n* = 2); and (iv) a positive margin in the secondary neurovascular bundle resection (*n* = 2). In 49 of 64 patients with positive IFS surgical margins, a secondary resection had been performed, which led to conversion to a definitive negative margin on the ipsilateral neurovascular bundle side in 47 (95.9%) patients; one patient had a positive surgical margin in the secondary resection.

**Table 3:** Final pathological characteristics after radical prostatectomy

Parameters	NeuroSAFE patients	Unilateral or bilateral positive frozen section(s)
Number (%) of patients	258	64 (24.8)
Grade Group, *n* (%)		
1	26 (10.1)	3 (11.5)
2	115 (44.6)	34 (29.6)
3	88 (34.1)	22 (25.0)
4	12 (4.7)	1 (8.3)
5	17 (6.6)	4 (23.5)
Tumour stage (pT), *n* (%)		
pT2	140 (54.3)	31 (22.1)
pT3a	79 (30.6)	22 (27.8)
pT3b	39 (15.1)	11 (28.2)
Positive surgical margin, *n* (%)		
pT2	35 (25.0)	
pT3a	33 (41.8)	
pT3b	21 (53.8)	

The median operation duration was 194 min and decreased over time (Spearman rho −0.18; *P* = 0.005). No significant difference was found between unilateral and bilateral NeuroSAFE procedures. The median time for the first 100 NeuroSAFE procedures was 203 min (IQR 175–229 min), and that for the last 100 NeuroSAFE procedures was 189 min (IQR 163–212 min) (*P* = 0.03).

## Discussion

Extensive IFS analysis according to the NeuroSAFE procedure enables more frequent nerve‐sparing surgery without having a negative impact on oncological outcome.^13^ In the current study, 74 of 491 (15.1%) NeuroSAFE specimens from 64 patients had positive surgical margins. In 39 of 54 (72.2%) secondary resections performed because of a positive NeuroSAFE finding, no remaining tumour was present in the neurovascular bundle. The positive IFS surgical margin length was significantly smaller in these cases, and more often present in only one slide. No tumour was present in secondary resections when the IFS surgical margin was positive in one section with a length of ≤1 mm and Gleason pattern 3. These results indicate that secondary neurovascular bundle resection might be omitted in cases with limited low‐grade disease at the positive IFS surgical margin.

Our positive predictive value of 85.1% and our negative predictive value of 95.4% for NeuroSAFE analysis are well in line with those reported by others.^13^, ^15^, ^18^, ^19^ Also, our proportion of 27.8% tumour in the secondary neurovascular bundle resections corresponds well with the proportion of 23% reported by Schlomm *et al*. in 1368 cases, and the proportion of 33% reported by Fromont *et al*. in 24 cases.^13^, ^20^ In contrast, Mirmilstein *et al*. found a higher proportion of 42.4% tumour in secondary resections among 33 resections.^15^ Our sensitivity of 76.8% is lower than those reported by Schlomm *et al*. (93.5%) and Mirmilstein *et al*. (90%), but higher than that reported by Tsuboi *et al*. (62%), whereas our specificity of 97.3% is in line with previous studies.^13^, ^15^, ^21^ As the proportion of tumour in secondary resections was comparable to that in other studies, our lower sensitivity might be explained by differences in the local work‐up of corresponding FFPE blocks, or the use of more strict criteria for calling a positive surgical margin.^13^, ^20^

If no tumour was found in the secondary resection, the positive IFS surgical margin length was mostly small. Although positive surgical margin status is not equivalent to biochemical recurrence, recurrence rates do increase with incremental cumulative length and tumour grade in the surgical margin.^22^, ^23^, ^24^, ^25^, ^26^ Several studies found that patients with a positive surgical margin length of ≤3 mm had similar biochemical recurrence‐free survival as those with negative surgical margins.^22^, ^26^, ^27^ Furthermore, the presence of Gleason pattern 3 in the surgical margin has been associated with a decreased risk of recurrence.^28^, ^29^

The introduction of NeuroSAFE requires standardisation of work‐up, evaluation, reporting, and clinical decision‐making. In our study, none of the patients with a positive intraoperative surgical margin in one tissue section, with a length of ≤1 mm, and Gleason pattern 3 into the ink had tumour in the secondary resections. On the basis of the above‐mentioned RP studies and our preliminary data, our group decided to abstain from secondary neurovascular bundle resection in cases meeting these criteria. Intraoperative reporting of positive NeuroSAFE procedures in our centre therefore routinely includes the following parameters: location as determined by distance from the apex, number of positive slides, cumulative length, and Gleason pattern in the margin. This synoptic reporting allows for standardisation of subsequent intraoperative decision‐making, and serves quality assurance purposes.

Extensive IFS techniques are increasingly being applied in European prostate cancer operation centres. Other groups have shown that NeuroSAFE results in an increased amount of nerve‐sparing surgery, but randomised controlled trials in relation to functional outcome are still ongoing.^13^, ^15^, ^26^, ^30^ Despite its putative positive effects on functional outcome, the NeuroSAFE technique requires logistic adaptations in pathology laboratories for processing and reporting of 10–20 frozen sections per RP. In our centre, this was achieved by a team of three technicians who simultaneously prepared frozen sections at two cryostat stations. After the first 100 NeuroSAFE procedures, the median time for NeuroSAFE processing and reporting decreased from 46 to 41 min. This time is comparable with that of Beyer *et al*., who reported an average NeuroSAFE pathology time of 35 min in >1000 patients, indicating that optimisation is still possible with larger numbers of procedures.^14^

This is the first detailed study on the pathological evaluation of IFS according to the NeuroSAFE technique, and might serve as guidance for centres introducing this procedure. However, the number of patients was relatively limited, and the change of protocol with regard to intraoperative decision‐making may have caused a bias. Furthermore, follow‐up was too short for analysis of oncological or functional outcome in our cohort.

In conclusion, this study provides guidance for reporting and clinical decision‐making for intraoperative NeuroSAFE procedures. In patients with positive surgical margins of ≤1 mm in one section with Gleason pattern 3, secondary nerve‐bundle resection might be omitted, leading to maximisation of nerve‐sparing prostate cancer operations.

## Conflicts of interest

The authors declare no conflicts of interest.

## Author contributions

M. A. van der Slot, M. A. den Bakker, M. Kliffen, N. N. T. Goemaere, L. M. Budel, K. E. Hamoen, S. Klaver, M. B. Busstra, J.B.W. Rietbergen, M. Gan and M. J. Roobol performed the research. G. J. L. H. van Leenders, M. A. den Bakker, C. H. Bangma and M. J. Roobol designed the research study. J. Helleman, M. J. Roobol and C. H. Bangma contributed essential tools. M. A. van der Slot, M. A. den Bakker and G. J. L. H. van Leenders analysed the data. M. A. van der Slot and G. J. L. H. van Leenders wrote the paper.
